# Collaborative Musical Creativity: How Ensembles Coordinate Spontaneity

**DOI:** 10.3389/fpsyg.2018.01285

**Published:** 2018-07-24

**Authors:** Laura Bishop

**Affiliations:** Austrian Research Institute for Artificial Intelligence (OFAI), Vienna, Austria

**Keywords:** creativity, ensemble performance, embodiment, emergence, mental imagery, communication

## Abstract

Music performance is inherently social. Most music is performed in groups, and even soloists are subject to influence from a (real or imagined) audience. It is also inherently creative. Performers are called upon to interpret notated music, improvise new musical material, adapt to unexpected playing conditions, and accommodate technical errors. The focus of this paper is how creativity is distributed across members of a music ensemble as they perform these tasks. Some aspects of ensemble performance have been investigated extensively in recent years as part of the broader literature on joint action (e.g., the processes underlying sensorimotor synchronization). Much of this research has been done under highly controlled conditions, using tasks that generate reliable results, but capture only a small part of ensemble performance as it occurs naturalistically. Still missing from this literature is an explanation of how ensemble musicians perform in conditions that require creative interpretation, improvisation, and/or adaptation: how do they coordinate the production of something new? Current theories of creativity endorse the idea that dynamic interaction between individuals, their actions, and their social and material environments underlies creative performance. This framework is much in line with the embodied music cognition paradigm and the dynamical systems perspective on ensemble coordination. This review begins by situating the concept of collaborative musical creativity in the context of embodiment. Progress that has been made toward identifying the mechanisms that underlie collaborative creativity in music performance is then assessed. The focus is on the possible role of musical imagination in facilitating performer flexibility, and on the forms of communication that are likely to support the coordination of creative musical output. Next, emergence and group flow–constructs that seem to characterize ensemble performance at its peak–are considered, and some of the conditions that may encourage periods of emergence or flow are identified. Finally, it is argued that further research is needed to (1) demystify the constructs of emergence and group flow, clarifying their effects on performer experience and listener response, (2) determine how constrained musical imagination is by perceptual experience and understand people's capacity to depart from familiar frameworks and imagine new sounds and sound structures, and (3) assess the technological developments that are supposed to facilitate or enhance musical creativity, and determine what effect they have on the processes underlying creative collaboration.

## 1. Introduction

Music performance is a social task. Most of the world's music is performed in groups, and even soloists are subject to influence from (real or imagined) audiences. Music perception is social too: audiences recognize social relationships and communicative behavior between members of a performing ensemble (Moran et al., 2015; Aucouturier and Canonne, 2017), and they infer human agency when hearing music–even without visual confirmation of a performer (Launay, 2015; Olsen and Dean, 2016), making sounded performances a means of interpersonal communication.

Music performance is also creative. In some musical traditions, new musical material is created via improvisation, while in others, sounded performances are created from loosely-defined visual notation. Performers across traditions adjust their playing to accommodate new performance environments as well as errors that result from imperfect technique (e.g., missed notes) or attentional lapses (e.g., missed repeats; Glowinski et al., 2016).

This paper addresses the question of how creativity is distributed across members of a music ensemble during performance. The focus is on processing that occurs online (i.e., during performance), though it is acknowledged that many offline musical tasks are creative as well (e.g., composing, structuring practice sessions, preparing an interpretation of a piece across successive rehearsals, evaluating other's performances, etc.). The real-time nature of music performance differentiates it from many other everyday tasks that require creative collaboration, such as brainstorming solutions to a problem with colleagues or jointly writing a report. There are also constraints on ensemble musicians' communication that do not exist in the context of many other tasks–for example, verbal discussion goes against performance conventions in many musical traditions, and gesturing may be hampered by the physical presence of instruments. For these reasons, music performance provides a particularly useful context for investigating how creative ideas emerge in real-time from the interactions between members of a group.

The structure of this paper is as follows. First, definitions of creativity and collaborative creativity in the context of music performance are outlined. I then present some theoretical perspectives on creativity and musical interaction that conceptualize creativity as distributed between interacting individuals and their social and material environments, and I argue that a refined framework drawing on these ideas is needed to guide ongoing research efforts. Next, some of the key mechanisms that are thought to support collaborative creativity in music ensembles are outlined, including flexible and persistent idea generation, musical imagination, communication, and empathetic attunement. The concepts of flow and emergence, which are central to discussions of collaborative creativity, are then considered, and some of the conditions that encourage group flow are discussed. Finally, some topics that are important to address in future research are identified.

## 2. Creativity

Creativity describes the component of human cognition that enables generation of output (an object, idea, performance) that is both novel and significant (Dietrich, 2004). In research contexts, creative output is typically evaluated on the basis of its originality and appropriateness. In artistic domains such as music performance, negotiating a balance between originality and appropriateness means maintaining flexibility within a given set of stylistic constraints. It is important not to confuse creativity with either originality, defined as the degree of novelty of a creative output relative to a given sample of related outputs, or value, the quality assigned to a creative output by a receiving audience (Williamon et al., 2006). Creativity is a component of cognition, while originality and value are evaluations made by others in the context of their own cultural experiences.

Recent theoretical frameworks include this evaluation process as a critical component of the overarching creativity construct. Fischer et al. (2005) describe four components of creativity: (1) originality, (2) expression, the externalization of the creative idea, (3) social evaluation, the process by which others consider the creative output and judge its value and (4) social appreciation, the process of encouraging or discouraging further creative efforts. As discussed in the next section, current theories endorse the idea that creativity does not function in a vacuum or within the confines of an individual mind, but rather, is shaped continually, in real-time, by past, present and anticipated interactions with the external world. This view is in contrast to earlier work on creativity, which focused on the internal cognitive processes of individuals, and treated these processes as separable from external influences.

### 2.1. Collaborative creativity: forms and levels

Collaborative creativity refers to the distribution of creativity across members of a group as they collaborate to solve a shared problem. It is in contrast to a division of labor, where each group member is assigned a part of the task and the collective outcome is equal to the sum of individual contributions. Collaborative creativity involves more complex interaction between group members and can yield an outcome that is greater than the sum of individual contributions. This greater collective outcome arises because the difference in task conditions prompted by group members working together, instead of individually, allows for the occurrence of ideas that cannot be attributed to any one person–a phenomenon referred to as emergence (see section 4; Fischer et al., 2005).

Creative collaboration between people can be (1) serial, if an individual creates something in isolation, then presents their creation to others who can build on it, (2) parallel, if group members create things separately, then bring them together to combine them into something new, or (3) simultaneous, if group members create something together, at the same time (Fischer et al., 2005). Simultaneous collaboration is of primary interest to the current discussion, though serial and parallel collaboration can be observed in the context of music performance as well. In particular, rehearsal of ensemble music often involves parallel collaboration, as ensemble members may do preliminary preparation of their own parts of a piece before playing it together as a group.

Seddon and Biasutti (2009) observed a professional string quartet and a student jazz sextet in rehearsal and noted three levels of interaction between ensemble members: instruction, which occurred when one group member communicated to another what to do; cooperation, which occurred when group members communicated to ensure that output was cohesive; and collaboration, which occurred when group members took creative risks, leading to the emergence of something new. Of particular interest to the current discussion is how performers move from the level of cooperation to the level of collaboration: what conditions prompt or prevent a higher level of interaction? How do interactions aimed at cooperation and interactions aimed at collaboration differ? These are among the questions explored in the later sections of this paper.

### 2.2. Creativity as embodied and distributed

Contemporary theories of creativity incorporate ideas from distributed cognition and dynamical systems theory, emphasizing the role of the social and material environments in which creative processes are carried out (Schiavio and Høffding, 2015; Linson and Clarke, 2017). This is in contrast to early studies of creativity, which focused on individuals' internal cognitive processes. This section of the paper discusses three theoretical approaches–“5 A's” creativity framework, the extended mind thesis, and the embodied music cognition paradigm–and how these approaches might be applied to an explanation of collaborative creativity. The aim is to define a theoretical conceptualization of collaborative creativity as involving a network of interactive, embodied, socially-situated, and externalizable processes.

Proposed by Glǎveanu (2013), the “5 A's” creativity framework defines five components: (1) Actor(s) who engage in (2) Actions (i.e., creative thinking externalized) that bring about an (3) Artifact (creative output) in the context of (4) an Audience (the social environment) and (5) Affordances (the material environment). This framework is a reworking of the earlier “4 P's” framework (comprising Person, Process, Product, and Press; Rhodes, 1961), re-designed with the aim of emphasizing the interdependence of the five components (whereas the “4 P's” were conceptualized as separable, and often studied independently).

According to this theory, interaction between the actor(s) and audience is critical, as it is the audience, who, presented with output produced by the actors, determines it to be creative (Csikszentmihalyi, 1999). That is, the creative quality of the actor's output is not realized until others have recognized it as such. This emphasis on evaluation places the audience in almost as important a role for achieving creative output as the actors (Glǎveanu, 2013). It should be noted that an audience may be real and present, providing live feedback to the actors (e.g., as when people attend a concert) or imagined/anticipated, in which case the actors may become the audience vicariously by assuming an audience perspective (e.g., when students consider how a performance might be received by the judges at their upcoming exam). During collaborative creativity, individual collaborators can be said to fulfill the roles of actor and audience simultaneously, as they are continuously judging the creative quality of each other's output while also producing creative output themselves. The extent to which collaborators judge each other's output to be creative can either encourage or discourage their own continued participation in the task and influence their willingness to take creative risks.

Also emphasizing the interdependence of actors, actions, and the environment, the extended mind thesis proposes that some cognitive processes are partially composed of actions made within the “we-space,” a dynamically structured physical space surrounding a person in which interaction with others is possible (Krueger, 2010). Multiple levels of we-space exist: personal space is taken up by the body; peripersonal space immediately surrounds the body and is accessible via auditory, visual, and tactile perception; and extrapersonal space is beyond the person's immediate reach and accessible only via auditory and visual perception. Gestures are an important means of navigating interactions in the we-space; they are an externalization of the gesture giver's cognitive-affective processes and involved in driving those processes, while simultaneously facilitating the task of the gesture receiver by narrowing the range of responses they have to choose from.

Interpersonal coordination in the we-space is thought to be partially a process of co-regulation, or continuous adaptation to one another's expressive behavior (e.g., automatic mimicking of facial expressions during conversation). Co-regulation distinguishes “focused interaction” (i.e., collaboration with a shared focus of attention) from “unfocused interaction” (i.e., co-presence without shared attention). For example, members of a music ensemble may adapt to each other's behavior during collaborative performance, which constitutes focused interaction, but not while practicing individually in the same rehearsal space, which constitutes unfocused interaction. The idea of co-regulation is much in line with the idea of coordination emerging dynamically from pre-reflective interactions at the level of body movement, which has been discussed in the music cognition literature (e.g., Maes, 2016). This idea is explored in greater depth in Section 3.3.2.

Like the “5 A's” and extended mind frameworks, the embodied music cognition (EMC) paradigm conceptualizes cognition as distributed between a person's brain, body, and environment. The body is thought to mediate interactions between subjective experiences and the external world, during both music performance (as meaning is transformed into sound) and music perception (as meaning is constructed from sounded stimuli; Leman and Maes, 2014; Maes et al., 2014; Moran, 2014). Perceptual-motor coupling has been proposed as a possible mechanism for body-mediated meaning formation. Perceptual-motor coupling occurs when perceptual events and the motor commands needed to produce them share overlapping neural representations. This shared coding creates an association that can be activated bidirectionally–actions can prompt expectations for specific perceptual effects, and perceived or anticipated effects can prime related actions (Prinz, 1990; Jeannerod, 2003).

The EMC paradigm posits that during group interaction, perceptual-motor coupling functions at multiple levels simultaneously (Leman, 2012; van der Wel et al., 2016; MacRitchie et al., 2017). At a lower level, motor activity and sensory input are in continuous interaction, enabling automatic regulation of performance technique and entrainment between ensemble members. At a higher level, performers draw on a repertoire of learned gestures to control their own playing and achieve more deliberate coordination with co-performers. In Section 3.3, these coordination modes are discussed in greater depth.

The theoretical perspectives outlined here suggest that a music ensemble should be thought of as a system in which all components, including individual performers, their instruments, the audience, the performance space, are interdependent and dynamically interacting. Empirical study of the processes underlying ensemble performance increasingly reflects this perspective. Still, most research thus far has focused on the processes involved in achieving and maintaining interpersonal synchronization in situations with relatively high temporal predictability (Keller and Appel, 2010; Loehr and Palmer, 2011; Ragert et al., 2013; Repp and Su, 2013; Zamm et al., 2014). To a large extent, designing controlled experimental conditions has meant reducing the demands on performer's creativity as much as possible. Whether the mechanisms that underlie performance on such controlled tasks generalize to performance under normal conditions – when the demands on creativity are high–is unclear. As discussed in the next section, it seems likely that additional mechanisms must be activated for ensembles to play coherently under conditions that demand creativity.

## 3. Mechanisms for musical creativity

Creative accomplishments are seen in a wide range of domains, from everyday problem-solving to interpersonal to artistic to scientific. The question of how domain-general and domain-specific processes combine to support performance on creative tasks is still an open question. Exceptional creative ability within a given domain usually depends on a person having extensive domain-specific knowledge and, in some cases, specialized motor skills (Ericsson, 1998). On the other hand, neuroimaging studies have shown that while the patterns of brain activation seen in people engaged in creative behavior are largely task-specific, certain regions (specifically, the lateral prefrontal cortex, inferior parietal cortex, and lateral posterior temporal cortex) are activated consistently regardless of the task (Gonen-Yaacovi et al., 2013). These regions may support a general network of creative abilities.

Barbot and Tinio (2015) argued that while evidence of a unitary, domain-general creativity capacity (i.e., similar to the “g” factor of intelligence) is limited, there seems to be a set of general creative resources that combine in different ways to support performance on a range of tasks. These resources include different processing strategies, such as associative thinking, selective combination, perseverance, and elaboration, as well as general intelligence, motivation (An et al., 2016) and mindset (Bittner and Heidemeier, 2013). Creative performance on a given task is facilitated when an optimal combination of resources is drawn upon. There is likely to be an ideal “fit” between the resources that are activated and the demands of the situation. For instance, De Dreu et al. (2011) found that trait behavioral activation–the tendency to carry out goal-directed behavior and respond with positive feelings to signs of an impending reward–potentiates creativity on tasks that afford flexible and global processing, but impedes creativity on tasks that afford local processing.

In performing creatively, ensemble musicians face two primary challenges: generating original (but stylistically appropriate) ideas and maintaining coordination while translating these ideas into musical output. Meeting these challenges draws on a large network of cognitive processes, likely a combination of general and task-specific. Three processes proposed to be central to creative collaboration are highlighted in the current paper: potential mechanisms for generating ideas (spreading activation), elaborating and evaluating ideas (musical imagination), and coordinating the implementation of ideas (communication). These processes are discussed individually in the following three sections.

Much of the research referenced in these sections—especially in relation to idea generation and musical imagery—adopts an individualistic perspective, focusing on cognitive processes within individuals. This is in contrast to the theoretical perspective endorsed in this paper, that collaborative creativity is embodied and distributed. Currently, little of the published research on creativity focuses on collaboration, and a theory of collaborative creativity has not yet been proposed. Therefore, this paper aims to identify theoretical concepts and empirical observations from individual-focused research that may be applicable to collaborative contexts, and highlight gaps in what these theories and and observations are able to explain.

### 3.1. Flexible and persistent modes of idea generation

Theories of creativity commonly distinguish between two contrasting processing modes – cognitive persistence and cognitive flexibility (Dietrich, 2004; Nijstad et al., 2010). Cognitive persistence involves sustained attention and controlled, incremental, and structured exploration of ideas. Cognitive flexibility, in contrast, involves divergent thinking, a global focus, the use of broad cognitive categories, and frequent switching between categories.

Dietrich (2004) defines four subtypes of creative processing by crossing flexibility and persistence (which he refers to as spontaneous and deliberate modes of thinking) with cognitive and emotional knowledge domains. Most creative tasks are said to engage a combination of these modes. For example, Dietrich suggests that creativity in the arts derives from emotional responses to environmental stimuli, and that artistic inspiration is thus largely the result of a flexible-emotional mode of processing. It should be added that artistic creativity can also draw substantially on cognitive knowledge domains (e.g., music theory, mathematics) and cognitive persistence (especially in non-real-time tasks, e.g., composition). Creative insights–defined as the conscious realization of an idea in working memory–can occur via any of the processing modes (Dietrich, 2004).

More recently, the dual pathway to creativity model was developed, positing the existence of persistence and flexibility pathways (Nijstad et al., 2010; De Dreu et al., 2011, 2012). The “persistence pathway” is critically supported by working memory, while the “flexibility pathway”, characterized inhibition, defocused attention, and automatic spreading of activation, is only minimally dependent on working memory. The authors behind the dual pathway model posit a role for emotion in creative performance, arguing that performance on any type of creative task can be influenced by the performer's emotional state. Both trait (personality associated) and state (temporarily activated) related mood characteristics are thought to mediate processing along persistence and flexibility pathways (De Dreu et al., 2008; Nijstad et al., 2010). “Activating” moods that are positive in tone (e.g., happiness) can improve creative performance by promoting cognitive flexibility, while activating moods that are negative in tone (e.g., fear) improve creative performance by promoting cognitive persistence. “Deactivating” moods (low in arousal; e.g., relaxation, sadness) seem to offer no such benefit for creative performance.

Schubert (2012) proposed a model for musical creativity that explains how processing might proceed along the flexibility pathway, in particular–though a similar explanation might also describe processing along the persistence pathway. Based on spreading activation theory, the model posits that activation spreads between nodes – which are abstract units representing knowledge and emotions–via links representative of learned associations.

Schubert describes the process of spreading activation as automatic, proceeding with or without conscious attention, and suggests that creative inspiration occurs when new paths form spontaneously between previously unconnected nodes. The process of spreading activation is driven by a desire to activate “pleasurable” nodes and inhibit “painful” nodes. As an example, improvisation involves constructing new musical sequences that fit within a given framework: musicians are guided in this task by the pleasure that comes from alighting upon ideas for patterns that fit, while simultaneously avoiding patterns that break from the framework. Central to Schubert's model is the idea that maintaining positive feelings is a critical component of musical creativity. As a potential extension to the model, it might be argued that processing along the persistence pathway involves controlled, incremental exploration through the network of nodes, driven by the same desire to achieve pleasing results.

The models presented in this section were developed to explain creative performance on individual, not collaborative tasks. How generalizable are these ideas to collaborative situations? The pool of cognitive resources available to a group is greater and more varied than would be the case for individuals performing a similar task alone. As a result, there is the potential for a wider variety of associations between ideas to be made. This could lead to more creative performance – but it could also lead to a lack of cohesion between outputs. There is also potential for conflicts to arise within the pool of cognitive resources that either facilitate or impair the creative process. For example, if members of an ensemble were to have different concepts of how a performance should progress (i.e., how the structure should unfold), then in terms of Schubert's spreading activation model, an idea that is “pleasurable” for one performer might be “painful” for another. Further investigation of how ensemble members negotiate specific, open-ended problems (e.g., interpretation of particularly ambiguous passages of a new piece) might clarify how conflicts are resolved and facilitate development of a model that accounts for collaborative creativity.

### 3.2. Using musical imagination to elaborate and evaluate ideas

The idea of spreading activation is closely linked to the idea of imagery. Indeed, they could be said to describe two parts of the same process: spreading activation is the mechanism through which nodes in a knowledge network are selected, and imagery is the activation of those nodes in memory. This section of the paper discusses the potential role of musical imagery in facilitating the search, selection, and evaluation of ideas during music performance.

Musical imagination has been suggested to underlie creativity in both music perception and performance (Hargreaves, 2012). *Musical imagination* refers to the human capacity to experience music in a way that is not a direct and immediate consequence of having perceived it. In the current paper, the term *musical imagery* is used to refer to the process of experiencing music in this way. While musical imagery has traditionally been defined as a form of mental imagery, I will avoid characterizing it as a specifically and exclusively mental process, as it might also be said to involve activation of the motor system, even if no overt movement is apparent (Aleman and Wout, 2004; Chen et al., 2008; Bernardi et al., 2013a; Bishop et al., 2014).

Musical imagery involves the multimodal activation of musical knowledge and the (re-)construction of musical stimuli in working memory. It is to be distinguished from the process of remembering details about music: recalling that Rachmaninoff's Piano Concerto No. 3 begins in the key of D minor is different from imagining the sound of the first chords or the feel of playing the piano line. The pitch (Aleman et al., 2000), timing (Janata and Paroo, 2006; Jakubowski et al., 2016), dynamics (Wu et al., 2011; Bishop et al., 2014), and timbre (Halpern et al., 2004) of perceived music can be imagined with high veridicality. Emotion is also perceived similarly in sounded and imagined music (Lucas et al., 2010).

Musical imagery is sometimes–but not always–a controlled process, and people are sometimes–but not always–aware of it. It should therefore be described as a process that is accessible to attention. Sometimes mental images are the focus of attention; for instance, during mental rehearsal (Bernardi et al., 2013b; Bach et al., 2014) or when distracted by an earworm (Müllensiefen et al., 2014; Floridou et al., 2017). Such instances are most likely to occur offline (i.e., not concurrent with overt performance, though still evolving in real-time). Online, many concurrent processes compete for a performer's attention, so even though imagery can be an important part of the action planning process, it might proceed largely without the performer's awareness (Keller and Appel, 2010; Bishop et al., 2013). Musical imagery is less often referred to in the context of music perception, but can nonetheless be said to contribute. Sounded music unfolds over time, and listeners must maintain some evolving representation of it in memory in order to make sense of the structure. Evidence of this process can be seen in the way listeners are able to re-interpret previously-established tonal contexts when incongruous chords are added to a progression (Bailes et al., 2013).

If musical imagery involves re-activating musical knowledge in memory, what is its relation to creativity, defined as the generation of something new? In other words, what is the difference between creative imagery and recall? Benedek et al. (2014) examined brain activity while people were engaged in a divergent thinking task (the alternate uses task), and observed different patterns of activation during recall of known ideas and generation of new ideas. In particular, the generation of new ideas involved activation of the left inferior parietal cortex, which has previously been linked to imagery and mental simulation. Creativity on this task was demonstrated through the recall of known ideas and application of those ideas to a novel situation: giving “swing” as a possible use of a tire was considered a recalled idea, as participants had seen it before, while giving “picture frame” as a possible use was considered a new idea.

Imagery allows people who are engaged in creative tasks to evaluate the appropriateness and originality of activated ideas before expending energy in externalizing them. It may also play a critical role in the type of controlled and structured idea generation that is associated with the persistence pathway. As described above, the persistence pathway draws on working memory: performance on tasks that encourage controlled generation and evaluation of ideas has been shown to suffer under high cognitive load conditions (De Dreu et al., 2012). This study also showed that cellists with high working memory capacity performed increasingly creative improvisations across several trials, while cellists with low working memory capacity performed decreasingly creative improvisations. The authors suggest that improvisation requires a great deal of planning and mental structuring, especially in cases where several rounds of improvisation will be required, and a high working memory capacity helps with maintaining a representation of that structure. When musical ideas are maintained in working memory (i.e., imagined attentively), they are accessible for reflection and evaluation. Musicians may, therefore, use imagery to structure their search for creative ideas and reflect on possible outputs.

In addition to its roles in idea generation and evaluation, musical imagery allows for manipulation of recalled material without interference from externalized sounds or movements. Composers, in particular, report using imagery to evaluate and elaborate on their ideas. Some claim that this is critical to do before trying to translate those ideas to an instrument or score, as creative thinking becomes more constrained and ideas become harder to change after that point (Agnew, 1922; Bailes and Bishop, 2012). For performers, the process of deliberately manipulating or elaborating on images often occurs offline (e.g., during mental rehearsal, or when deciding how a piece should sound).

Online, there is not usually time to imagine different variations of an idea before implementing it. However, the malleability of musical images – the fact that they can be disrupted by incoming signals or deliberately manipulated – may be critical for creative performance. This malleability may help performers to be flexible in their playing, allowing them to adjust for errors (Glowinski et al., 2016) and accommodate new ideas in real-time (either their own or their co-performer's). The use of anticipatory imagery as a means of guiding musical performance has been studied empirically (Keller and Appel, 2010; Keller et al., 2010; Bishop et al., 2013) and described anecdotally by highly skilled musicians (Trusheim, 1993). Anticipatory imagery involves activating evolving expectations of how musical output should sound, feel, and/or look, and facilitates selection of the action parameters needed to achieve the desired output by way of inverse perceptual-motor activation (see perceptual-motor coupling in section 2.2). That these expectations are accessible to attentive reflection (even if not always attended to) is important: this feature of the imagery process enables deliberate revision of plans as well as constant monitoring of performance success.

As stated above, imagery contributes to listeners' abilities to make sense out of musical performances as they unfold over time. This role of imagery in music listening is central to ensemble performance, because a large part of the task of performing with a group is listening to and taking cues from each other. Inter-performer communication is discussed in the next section of this paper, but here, I want to emphasize how important it is for ensemble musicians to listen to each other with “open ears” in order to perform creatively. That is, while hearing the combined output of the group, they must be open to receiving new ideas, changing their interpretation of already-performed structures, and pursuing deviations from the prescribed script that is guiding their performance. This openness requires a strong awareness of the group's current and previous output, which, I would hypothesize, takes the form of a flexible guiding image.

### 3.3. Communication drives alignment of ideas

The term “communication” refers broadly to the transfer of information that occurs between members of a group. Communication between ensemble members can take many forms: fluctuations in audio signals produced by an instrument, audible breathing, shifts in eye gaze, changes in posture, overt gestures, or facial expressions. The information that is transferred might relate to performer's interpretation of the music, their engagement in the task, a shift in roles, or an acknowledgment of a mistake, among other things.

Some communication between musicians is necessary for ensembles to perform coherently. This is clearly shown by studies testing musicians' success at playing with disrupted communication channels—while eliminating visual communication between performers has relatively minor effects, eliminating audio communication leads to substantial temporal misalignment (Bishop and Goebl, 2015). Delays in audio communication likewise impair coordination, even rendering performance non-interactive if the delays are large enough (Bartlette et al., 2006). Here, research that has been done on communication in music ensembles is considered, along with some criticisms of the assumptions that underlie this research and some recent studies that attempt to test these assumptions.

#### 3.3.1. Sharing intentions: simulation and prediction

Widespread in the literature on ensemble performance—and in the broader literature on joint action—is the idea that collaborating members of a group each have individual intentions regarding their own contribution to a task, as well as shared intentions regarding how their individual contribution will fit into the group's combined output (e.g., Keller, 2001). Performers' *intentions* encompass their action-oriented anticipatory imagery as well as knowledge relating to the expressive constructs that they plan to implement. The intentions performers have are multi-leveled and exist in parallel across overlapping time scales. High-level intentions, which span a long time frame, might relate to the overall structure of the performance (e.g., formal structure as notated in a score) or general expressive content. In contrast, low-level intentions, which are directly involved in action planning, unfold rapidly and often without conscious control. Coordinating a joint performance successfully requires individual performers to share clues to their own intentions while also monitoring the signals given by others.

Musicians' low-level intentions are often studied by manipulating the expectedness of the sounds that their movements generate. When manipulated sound output induces performance errors or other compensatory behavior, we conclude that those manipulations were not in line with the musicians' intentions, and that their action planning system is trying to correct for the “error.” Responses to unexpected sound output can also be observed in readings of brain activity. Research using these methods has shown that when playing duets, pianists anticipate the sounds of their own and their partner's key-presses, as well as the combined output (Loehr et al., 2013). For instance, novice pianists who learn to play a simple melody with live accompaniment perform better at test with accompaniment than without, suggesting that they learn their own melody in terms of how it fits into the combined output (Loehr and Vesper, 2016).

Action simulation is thought to underlie musicians' anticipation of others' sound output during music performance (Jeannerod, 2003). This process of covert action representation engages coupled perceptual-motor brain networks without necessitating overt movement (Patel and Iversen, 2014). Simulation is facilitated when the action and its resulting sound are strongly coupled in the brain. More effective simulation leads to better anticipation and improved temporal coordination between performers (Keller et al., 2007; Wöllner and Cañal-Bruland, 2010).

Communication between performers is thought to support action prediction processes by providing cues to initiate the simulation process. While auditory communication in the form of a musical sound signal is usually sufficient for performers to maintain temporal coordination, they sometimes supplement their audio signals with visual signals (Badino et al., 2014; Kawase, 2014; Bishop and Goebl, 2017). Ensemble musicians are better able to predict the course of observed gestures when those gestures fall within their practiced repertoire (Wöllner and Cañal-Bruland, 2010; Bishop and Goebl, 2014), and better able to predict such gestures than are novice musicians (Luck and Nte, 2008; Petrini et al., 2009; Lee and Noppeney, 2014). It seems that for ensemble musicians, simulating co-performers' actions in response to a visual cue is a well-practiced task.

#### 3.3.2. Sharing intentions: when is it necessary?

The idea that successful ensemble performance necessarily involves performers communicating their individual intentions to each other and, ulimately, constructing shared intentions, has been a source of debate in the literature. Under some conditions, it is argued, coordination can emerge from local (often pre-reflective) responses to the gradually unfolding musical output, making a shared global plan and explicit communication unnecessary (Hutchins, 1990; Linson and Clarke, 2017). This is the perspective generally endorsed by the EMC approach (Schiavio and Høffding, 2015; Maes, 2016).

The description of coordination as emerging dynamically from local interactions is in line with musician's descriptions of group flow—as discussed in Section 4.2, group flow is characterized by joint feelings of effortlessness, a lack of self-awareness, and non-reflective patterns of thought. On the other hand, ensemble musicians also communicate with each other reflectively through overt body gestures and deliberate manipulation of sound output, particularly when working together to construct an interpretation of notated music (Williamon and Davidson, 2002; Davidson, 2012). Such evidence suggests that ensemble performance may be supported by different types of communication under different conditions (MacRitchie et al., 2017). Relevant to the current discussion is how much ensemble musicians draw on reflective and pre-reflective types of communication when performing naturally and creatively.

Ensemble musicians have been shown to exchange communicative gestures deliberately at critical moments in their performances, as a way of facilitating note coordination. Such cueing gestures often take the form of hand/arm movements or head nods (Bishop and Goebl, 2018). Breathing gestures are likely used as well and have the benefit of providing an audiovisual cue, but they are more difficult to measure experimentally. Cueing gestures can be exchanged at moments of sudden tempo or meter change (Kawase, 2014) or at piece entrances or re-entrances that require synchronization between performers (Bishop and Goebl, 2015, 2017). These are ambiguous, isolated moments when co-performer's expectations about how to play might not otherwise align. Performers might be expected to make greater use of communicative gestures during the early stages of rehearsal, when still unclear on how they would like the music to sound, than when performing well-practiced pieces. On the other hand, in some cases, gestures at structurally or expressively significant moments are retained through rehearsals and integrated into the performance script (Williamon and Davidson, 2002).

Visual communication between ensemble members may be particularly important when the demands on creativity are high, and a number of temporally ambiguous moments arise in the music. Note, however, that cueing gestures serve primarily to clarify irregular timing; whether or how they contribute to the coordination of other parameters remains unclear. Thus, greater use of visual communication during some types of creative performance (e.g., playing notated music with no meter) but not others (e.g., improvisation of temporally-regular music) might be expected.

Some recent studies, outlined below, have started searching for evidence that ensemble performance is supported—at least partially—by low-level interaction between members. These studies reject the assumption that coordination between ensemble members is necessarily dependent on the construction of shared intentions.

In an attempt to determine whether shared intentions are truly needed for a coordinated ensemble performance, some study has been made of collective free improvisation (CFI), a form of improvisation drawn upon in several musical genres. With other forms of improvisation, it is standard for performers to identify a framework to help structure their playing by reducing the range of possible contributions they could make. Such a framework, or “referent,” might include aspects of large-scale structure (e.g., in jazz, how many choruses to cycle through), melodic/harmonic content (e.g., themes, chord progressions, keys to use), leader/follower roles (e.g., a pre-arranged order of solos), and perhaps also some expressive content. Musicians engaging in CFI, in contrast, deliberately eschew the use of a shared referent, instead constructing musical structure in real time (Canonne and Garnier, 2015).

Canonne and Aucouturier (2015) tested for the presence of “shared mental models” (i.e., schemas) among musicians who regularly perform CFI. In particular, the hypothesis that musicians would have overlapping concepts of the CFI task and overlapping interpretations of certain musical elements was investigated. Musicians categorized musical excerpts from CFI performances based on how they would respond musically. Response similarity was calculated between participants and subjected to a nearest neighbor classification algorithm, which predicted familiarity between participants with higher than chance accuracy: musicians who performed together tended to interpret the musical excerpts similarly. Such a shared understanding of the music could (unintentionally) give collaborating musicians a common language with which to exchange ideas.

Pachet et al. (2017) tested the hypothesis that ensemble performance is partially driven by low-level interactions that emerge as relationships in the acoustic features of collaborating performers. These relationships are distinct from those that emerge as a result of performers adhering in parallel to a prescribed structural framework (“score effects”), and instead attributable to real-time interaction. A number of acoustic features were extracted from six improvised performances recorded by a five-member jazz bebop band, and comparisons were made between individual performers. No pair of features correlated reliably across performances, so even though significant correlations occurred within performances, the possibility that these were attributable to score effects could not be ruled out. As the authors point out, whether signs of pre-reflective interaction might be seen with higher-level information in performer's audio signals (e.g., rhythm patterns) is yet to be tested.

Interaction between performers might also emerge as relationships in features of their body movements—in particular, their ancillary body movements, or those not directly involved in sound production. Some studies of piano duet performance have shown evidence of coordination in patterns of pianists' head movements (Goebl and Palmer, 2009) and body sway (Keller and Appel, 2010). In a study by Ragert et al. (2013), pianists learned either one or both parts of piano duets, which they then performed for recording with another pianist, as their body movements were tracked. Pairs of pianists who knew both parts of the duets displayed a steady high degree of coordination in their body movements throughout the performances, while pairs who had learned one part were less coordinated at the start, but increased their coordination as the experiment progressed. The authors suggested that practicing both parts of a duet allowed pianists to construct a more thorough image of the piece structure, which facilitated timing predictions at the relatively long time scales at which head and torso movements unfold, improving movement coordination. This explanation implies that pianists intend, at some level, to coordinate their body movements, however, which may not be the case. An alternative explanation is that pairs of pianists who knew the full pieces were more likely to share an interpretation of it than were pairs who each knew a different part, and tended to display similar patterns of motion as a result of their overlapping interpretations.

On the other hand, a study by Badino et al. (2014) provides some evidence of ensemble musicians influencing each other's movements, indicating that coordinated patterns of ancillary movement can emerge as a result of performers' interactions. Head movements were tracked for members of a professional string quartet during performance under normal and perturbed conditions (in which the first violinist introduced unexpected expressive changes). Across takes, the first violinist exerted the strongest influence over the other musicians (measured with Granger causality), though his influence was reduced during the perturbation segments. Musicians' combined influence over each other was highest during technically complex sections of the piece, suggesting an increase in the communicative value of their movements during these sections.

The function of coordination in performers' ancillary movements is not clear. It could be an aesthetic aim, for the benefit of an observing audience, or meant to facilitate note coordination. It might also serve a motivational function by enhancing the feeling of interaction and engagement. Recent research on visual attention suggests that duo performers look at each other more often than we would expect if they were seeking only to clarify irregular timing (Bishop and Goebl, 2017). Instances of two-way eye contact also occur at predictable points in the performance, indicating that performers are not solely driven to look toward a “leader” for timing cues; instead, both performers monitor each other, perhaps as a means of communicating and confirming each other's engagement and understanding.

The literature described here paints a still-unclear picture of the nature of the communication processes that drive ensemble coordination – particularly the processes that drive the real time coordination of new and spontaneous ideas. The field is especially in need of further systematic study of low-level communication mechanisms. Until recently, it was difficult to capture low-level features of visual communication, in particular, in meaningful detail. However, developments in motion capture and motion analysis techniques–especially techniques that enable us to quantify the influence that collaborating musicians have over each other's movement patterns (e.g., Badino et al., 2014; Walton et al., 2017)–provide a promising means to understanding emergent coordination.

## 4. Emergence and group flow

In the Western classical music tradition, musicians prepare for public performances of a piece with extensive rehearsal and careful study of the score. Yet at the same time, they value creativity and spontaneity, as do their audiences (Repp, 1997b; Chaffin et al., 2007). A series of studies by Chaffin et al. (2006, 2007, 2010) have investigated how skilled musicians maintain enough control over their performances to be able to make spontaneous interpretive decisions, despite simultaneously drawing on highly automatized movements. The results of these studies suggest that creativity in performance depends on where musicians focus their attention. If attention is directed away from the music (e.g., focused on a distracting audience member or the performer's own anxiety symptoms), performance is likely to be automatic and uncreative; if attention is directed toward the music but focused on errors, performance is likely to be uncreative and cautious. Skilled performers seem to construct a structure of attention cues during rehearsal that relate to different aspects of expression and technique. These cues help musicians focus their attention during performance and allow for conscious interpretive decisions to be made.

For music ensembles, spontaneity in interpretation can manifest as emergence. Occurrences of emergence, along with group flow, seem to characterize ensemble performance at its peak. In this section of the paper, the concepts of emergence and group flow are addressed and conditions that encourage their occurrence are identified. In particular, an external focus of attention (i.e., toward the musical output and away from the self), which Chaffin has shown to be key for managing creativity in interpretive decisions, seems to be critical for achieving flow; likewise, shared knowledge of an intended guiding framework for the performance is thought to be important.

### 4.1. Emergence as a function of group interaction

Emergence, as defined in section 2.1, is a phenomenon that occurs when the collective output of the group amasses to greater than the sum of individual contributions. In some ways, ensemble performance is necessarily emergent, as individual contributions combine to form cumulative units with distinct structural meaning (e.g., three notes played by three performers combine to form a chord, which as a complete unit has meaningful harmonic implications that none of the three notes have independently). More relevant to the current paper, however, is emergence that corresponds to flexibility in interpretation of a prescribed structure (in the case of notated music) or the construction of substructures (in the case of improvisation within a set framework).

An alternate way of defining emergence is to say that it occurs when a group performs in a way that cannot be attributed to any one individual contributor. It can be argued that ensemble performance is not always emergent in this way. For example, social factors (e.g., skill level, age, position in a social hierarchy, etc.) or piece structure can combine to encourage performers to fall into leader/follower roles, which can result in one person making most of the interpretive decisions. Furthermore, ensembles do not always achieve what they set out to achieve, and while the goal might be a performance that is original and spontaneous, the outcome is sometimes poorly coordinated or uninspired.

A study by Hart et al. (2014), examined performance on the “mirror game” (Noy et al., 2011), a task for dyads that involves moving a pair of horizontal sliders back and forth along a track to create coordinated patterns of improvised movement. Periods of smooth, highly-synchronized motion emerged, which a subsequent study found to coincide with increases in heart rate and increases in correlation of heart rates between performers (Noy et al., 2015). Suggestive of emergence, these periods were less likely than other performance segments to carry the signatures of either performer's individual style. Notably, between-group overlap in motion characteristics was high, suggesting that these periods of emergent coordination were supported by predictable, rather than idiosyncratic, movement. As discussed in section 3.3.2, when necessary, performers can manipulate aspects of the audio and visual signals that they exchange to increase their predictability to each other.

The relationship of emergence to flow states, described below, is unclear. Is emergence more likely during periods of flow? Emergence in music performance, as defined in this paper, is potentially complicated to identify because it requires comparing the combined output of an ensemble to the output that individuals would produce if performing their part alone. A reliable method of quantifying differences between individual and group interpretations (e.g., similar to that used by Noy et al., 2011; Hart and Di Blasi, 2015) has yet to be defined. In future research, it will be necessary to investigate how often periods of emergence occur during ensemble performance, what prompts them, and how they shape audience members' perception of performance quality and expressivity.

### 4.2. External focus of attention and shared knowledge support group flow

Musicians sometimes find themselves in a state of acute absorption: wholly focused on the task of performing, they feel an intense connection to the music, which flows out seemingly effortlessly. This rare but rewarding experience is called flow, and is generally thought to arise from an optimal match between task demands and the performer's skills, which fuels a sense of intrinsic motivation (Keller et al., 2011). The concept of “flow” was originally identified at the individual level (Csikszentmihalyi, 1990), and has only more recently been found to occur at a group level (Sawyer, 2006). It is important to note that group flow is an emergent quality of groups engaged in creative performance; it is not reducible to study at an individual level, and it is not the same as individual flow in a group setting (Sawyer, 2006).

In a qualitative study involving interviews with regularly-performing (improvising) musicians, (Hart and Di Blasi, 2015) identified some common themes in musicians' descriptions of their flow experiences. Musicians described the conditions that they thought were necessary to build up to a state of flow, including being able to establish and maintain a sense of individuality and dismiss feelings of self-consciousness. Another theme that came through was the idea that flow states require a lack of awareness of the self and less reflective patterns of thought. The musicians spoke about not appreciating (reflecting on) the performance as it happens, and being unable to remember afterwards what they had played.

The term “mutual engagement” has been used to describe interperformer interaction during periods of group flow (Bryan-Kinns and Hamilton, 2009; Bryan-Kinns, 2013). Performers in this state are engaged with each other and with the music they are producing. Some conditions are posited to underlie performers' achievement of a group flow state, including a mutual awareness of each other's actions (i.e., who is contributing what and when), shared representations of the intended outcome, equal access to musical output, the possibility of modifying each other's output (e.g., by responding to it), and the possibility of communicating around the output (rather than exclusively through it; e.g., visually, through body gestures; Bryan-Kinns and Hamilton, 2009).

According to the Networked Flow model, group flow develops through three stages, which draw on successively higher levels of empathy (Gaggioli et al., 2013). Central to this model is the concept of social presence, described as an individual's ability to interact with others by understanding and sharing their intentions. At an initial stage, “proto-social presence” involves performers recognizing each other's motor intentions. The second stage, “interactive social presence,” involves each performer individually recognizing those intentions that are directed toward him/her. At the final stage, “shared social presence” involves performers entering into resonance with each other. Some support for the model was offered by a study of performance quality and self-report measures of group flow and social presence among rehearsing (3–7 member) bands. A positive relationship was observed between self-reported measures of group flow and social presence. Flow also related to self-ratings of performance quality, though not to expert ratings (Gaggioli et al., 2017).

A point of overlap between the Networked Flow model and the mutual engagement paradigm is the idea that shared knowledge of individual intentions is needed for group flow to emerge. What constitutes “intentions” is not entirely clear (see also section 3.3.2), but at a minimum, it is likely that performers must at least agree over the intended structure of the performance. Musicians' descriptions of their group flow experiences suggest that individual group members need to feel that they have a specific and valuable role to play–that is, they need to be able to conceptualize how their own contribution will fit into the collective outcome (Hart and Di Blasi, 2015). On the other hand, musicians may also benefit from having few constraints to limit the possible contributions that they can make (Canonne and Aucouturier, 2015). In a study by Walton et al. (2017), musical duos reported a greater sense of freedom when improvising over a drone backing track than when improvising over a swing bass line. They felt that the drone encouraged a greater degree of interaction. Indeed, their coordination in sound output and body movement was higher during improvisation over the drone.

In addition to shared structural intentions, a shared emotional state might also promote group flow. Seddon (2005) distinguished between sympathetic and empathetic levels of attunement, positing that sympathetic attunement between performers supports coordination of a cohesive performance, while empathetic attunement is necessary for flow states and the “spontaneous musical utterances” that characterize emergence (see also Seddon and Biasutti, 2009). Progression to empathetic attunement can be impaired by interperformer conflicts in musical style or skill. Empathetic attunement requires performers to assume each other's musical perspectives, and is therefore thought to draw on their capacity for empathy. Indeed, prior research has shown a correlation between duet performer's scores on measures of empathy and the strength with which they represent their co-performer's part (Novembre et al., 2012).

Empathy is defined on two dimensions: cognitive empathy relates to capacity for perspective-taking, while emotional empathy relates to the flow of feelings between people (Babiloni et al., 2011). The process by which emotional states spread from one person to another–called emotional contagion–is speculated to occur during creative collaboration, and could potentially help to support emergent coordination. Emotional contagion seems to occur between performers and listeners (Lundqvist et al., 2009) and empathy has been shown to mediate the process (Egermann and McAdams, 2013). However, whether this also occurs within performing ensembles has not yet been confirmed. In one study, ensemble musicians reporting on completed performances showed less overlap in their experienced affective states than in their perceptions of leadership (Morgan et al., 2015).

As a final point, group flow in music ensembles could be encouraged by a shared cooperative, rather than competitive, mindset. Outside the music domain, in the context of verbal divergent thinking tasks, the effects of cooperative vs. competitive mindsets are mediated by regulatory focus – that is, the tendency to attend to either promotion goals (aiming to achieve an “ideal self” through growth and development) or prevention goals (aiming to achieve an “ought self” by preventing failure; Bittner and Heidemeier, 2013). Activating a promotion focus seems to prompt people to adopt a cooperative strategy, which improves performance on the task, while activating a prevention focus prompts people to adopt a competitive strategy, which worsens performance. As a general rule, we can assume that most ensemble musicians intend to cooperate with their co-performers; however, it is possible that some performance situations prompt a prevention focus and/or an intention to compete. For instance, a student ensemble participating in a competition might be preoccupied with preventing technical errors or outperforming other groups, and in doing so constrain their own creative processes.

## 5. Research directions

Coordination is a broad and multilevelled construct. In this paper, the focus has been on coordination during creative performance – a high-level task that requires alignment of spontaneously-generated ideas in real-time, without prior practice. The processes that support lower levels of coordination (e.g., synchronizing periodic taps or regularly-timed duets) may be insufficient to explain the high-level coordination of creative ideas that ensemble musicians can achieve. I have highlighted some of the processes that could account for aspects of during ensemble performance. However, our discussion has raised a number of issues that are still relatively unexplored. Below, three lines of research are outlined that would benefit from further attention.

### 5.1. Explaining emergence and group flow

Further study of emergence and group flow will be critical to identify the mechanisms engaged by collaborative creativity during performance. Earlier, I made reference to some interview studies with ensemble musicians; these have been useful for obtaining descriptions of flow experiences from a first-person perspective, and have lent support to models that propose explanations for how flow and emergent coordination develop including models by Bryan-Kinns and Hamilton (2009) and Gaggioli et al. (2013). Largely absent from the literature, however, are systematic, empirical studies that test these models. We have some idea of the conditions that are necessary for group flow to develop, but what triggers the onset of a flow state? What conditions trigger emergence? How do group flow and emergence relate, and how these states maintained? How resistant is flow to perturbations resulting from technical errors or environmental disruptions?

At this stage, the answers to these questions seem largely theoretical. The literature might benefit from further efforts to manipulate potentially relevant factors and induce flow experimentally. The importance of musicians' focus (self-reflective vs. external) and interaction in real-time via auditory and visual channels might be tested this way. Focus could be manipulated by catering the instructions that performers receive: instructions that direct attention toward individual success or accuracy should encourage a self-reflective focus, while instructions that direct attention toward a particular expressive goal might encourage a external focus. Ideally, performances should be given under as naturalistic conditions as possible (e.g., established ensembles playing familiar under self-selected constraints). Flow would be best assessed using a combination of self-report, physiological, and behavioral (e.g., performance output, body movement) measures. In such a study, it would also be useful to analyse performance data for evidence of emergence; for example, by comparing solo and ensemble performances of the same material (e.g., as in, Hart et al., 2014; Noy et al., 2015).

Investigation of how group flow emerges during performance in non-Western musical traditions would also improve our understanding of the phenomenon–particularly in cases where performances are occasions for widespread participation, and there is not a strict performer/audience separation (Hill, 2012). In such cases, musicians may tend less toward a self-reflective focus than do musicians in Western traditions, who are often preoccupied with individual success and audience judgments (Hart and Di Blasi, 2015).

The relevance of musical imagery to group flow is still also a source of debate. As argued in section 3.2, imagery could facilitate flexibility during creative performance. According to Cochrane (2017), flexibility in performance means being able to choose between several responses to a given stimulus, and should only be possible for performers who can represent the possible responses before carrying one out. This should especially be the case when the musical structure is complex and requires sophisticated interpretation. Some authors have argued that imagery, or more generally, private intentions, are not necessary for ensembles to coordinate a cohesive performance (see section 3.3); however, it is unclear what other mechanisms could account for the flexibility seen in skilled performance. Cochrane (2017) goes on to explain how performers' intentions may critically underlie their flow experiences. While playing, performers monitor the disparities between their intended and output sound; disparities create a sense of tension, which is alleviated when the intended and output sounds match. The alleviation of tension enables a reduction in self-consciousness and perceived effort, allowing performers to focus on musical output in a way that is characteristic of flow. Thus, maintaining (and overtly realizing) intentions could enable the development of flow states. Further study would be needed to test this hypothesis.

Ultimately, many musicians could benefit from a clarified understanding of what causes group flow and how to encourage it. At present, research is still needed to identify the effects of flow states on musical output. As mentioned in section 4, Gaggioli et al. (2017) found that ensemble member's ratings of their own performance quality related to measures of group flow, while ratings of performance quality given by independent experts did not. Thus, the perception of success that motivates performers and fuels their sense of effortlessness may not relate reliably to the quality of musical output as perceived by an audience. On the other hand, audiences are sensitive to aspects of the interaction that occurs between ensemble performers. Aucouturier and Canonne (2017), for instance, showed that listeners use cues relating to temporal and harmonic coordination to decode social intentions (attitudes such as domineering, disdainful, or conciliatory) in improvised duo performances. Attentive audiences may pick up on evidence of group flow, and their perception or engagement with the performance might be enhanced as a result.

The literature would also benefit from more thorough investigation of the physiologial and social effects of flow. Physiologically, flow has been shown to share an inverted u-shaped relationship with stress-induced sympathetic arousal, and a positive linear relationship with parasympathetic heart rate control (Peifer et al., 2014). Cohen and Bodner (2018) observed a strong negative relationship between the occurrence of flow and performance anxiety among classical orchestral musicians, and suggest that devising means of encouraging flow might help reduce the effects of performance anxiety. Socially, some of the factors that support group flow, including joint attention (Wolf et al., 2015) and rhythmic synchronization (Hove and Risen, 2009), are also thought to underlie the heightened affiliation that has been shown to develop between musical partners. We might hypothesize that the bonding effects that are seen generally as a result of ensemble playing (Tarr et al., 2014; Pearce et al., 2016) are exaggerated in instances of group flow.

### 5.2. Recalling and creating: can we imagine what we have not perceived?

Though “free” because it is not driven by incoming stimuli, musical imagery is simultaneously constrained by the perceptually-shaped cognitive space in which it is carried out (Leman, 2001). As discussed in Section 3.2, imagery involves reconstructing elements of previously-perceived material. The process of reconstruction can be fairly accurate, yielding musical images that retain many of the parameters of the original percepts. Relevant to the issue of creativity, however, is the question of how free people are to manipulate or elaborate previously-perceived material. To what extent can people imagine what they have never perceived?

This question is particularly relevant to collaborative musical creativity, where, in optimal cases, the music that is produced is distinct from what individual group member would have produced alone (i.e., emergence occurs). As this paper has discussed, to achieve emergence in a collaborative performance, individual group members must be flexible enough to accommodate and elaborate on novel ideas. Sometimes – if the group includes members with vastly different musical backgrounds, or the musical genre encourages experimentation with sound and structure – the range of ideas that arise might be broad. In such cases, an ability to imagine musical structures (e.g., tone qualities, meters, pitch intervals) outside the performer's prior experience would be beneficial, if not critical.

It is important to note that the process of imagining music is an imperfect one, even if the aim is a precise reconstruction of a specific stimulus (Large et al., 1995; Dowling et al., 2002). Details of a musical experience can be erroneously perceived or encoded, or insufficiently embedded in a network of associations, making them difficult to retrieve. The use of heuristics and schemas in facilitating reconstruction can also lead to errors (Vuvan et al., 2014). Thus, people almost never imagine music precisely as it was perceived. More important for creative thinking is the ability that people have to selectively recall elements of prior perceptual experiences and recombine them into something new (“combinatorial play”). This is what we assume happens when musicians imagine a new improvisation or a new interpretation for a practiced piece: details relating to pitch, timing, instrumental tone, and dynamics are drawn from well-established networks of musical knowledge and re-assembled in a new way.

These images can then be manipulated. It was in the visual domain that evidence of “emergent properties” in imagery was first found–that is, evidence that images can be reinterpreted, allowing patterns to emerge that were not noticed at the time of perception. People can reinterpret simple geometric shapes in memory (e.g., identify new shapes formed by imagining a capital “H” superimposed on a capital “X”); complex shapes prove more difficult, probably because they require more resources to maintain in working memory in sufficient detail (Finke et al., 1989). In the musical domain, trained musicians using “notational audiation” to imagine music from a score are able to extract familiar melodies hidden in embellished phrases (Brodsky et al., 2003). Foster et al. (2013) tested musicians' abilities to imagine pitch and timing transformations (i.e., pitch transpositions and melody reversals) on simple melodies. The transformation task was found to activate parts of the bilateral intraparietal sulcus, a region that has been previously associated with visualspatial transformation and calculation.

Imagery may also facilitate translations between musical stimuli and sensations or perceived events. Music is an effective and versatile means of nonverbal communication, in part, because it activates so many associations for those involved in producing or hearing it. These associations often relate to emotion, and as such, the communication of emotion has received a great deal of attention in the literature (Juslin and Laukka, 2004; Molnar-Szakacs and Overy, 2006; Lundqvist et al., 2009; Lucas et al., 2010). Other constructs are known to be communicated as well, though, including sensations of motion (Eitan and Timmers, 2009; Olsen and Dean, 2016) and interpretations of musical structure (Clarke, 1993; Toiviainen et al., 2010). Even complex environmental events, such as animal behavior, changes in season, landscapes, or city life, can be communicated musically (without the aid of lyrics). Wong and Lim (2017) found imagery to facilitate children's creativity on a music composition task. Young children were instructed to construct audiovisual images of animals before composing short melodies in which the animals “came alive.” Scores of creativity (judged by experienced music teachers) were higher for participants in the imagery condition than for participants who did not receive imagery instructions. Thus, imagery may have helped participants translate between knowledge of animal characteristics and acoustic representations of those characteristics. Using imagery as a means of translation between modalities and representations arguably constitutes imagining what we have not perceived.

In sum, people have the ability to manipulate musical images in ways that deviate substantially from music they have perceived in the past. Whether people can create new tone qualities in their imagination (e.g., when constructing a new instrument or synthesizing a new sound) remains unclear. This is a question that should be addressed, especially given the increasing popularity of music that uses non-traditional methods of sound production or sound modification (e.g., electric guitars, synthesizers, digital musical interfaces, algorithm-based voices, etc.). Do musicians imagine the tone quality that they want to achieve before attempting to match it acoustically? Likewise, during group performance of music incorporating synthesized/digital sounds, how do performers adapt their sound to match (or compliment) their co-performers' sounds?

It will also be important to continue investigation of the motor aspects of musical imagery. Specifically, to what extent can people replicate in imagination what they have not previously performed (or do not know how to perform)? Section 3.3.1 discussed the role that motor simulation might play in interpersonal coordination. As ensemble musicians make increasing use of non-acoustic instruments and/or perform alongside algorithmically-controlled co-performers, will they draw on the same mechanisms for creativity and coordination as they do when playing acoustic instruments with human co-performers?

### 5.3. Does technology facilitate or constrain creativity?

Creativity is widely valued in Western music traditions. In some other traditions, this is not the case, and performers are instead expected to replicate the ideal performance of a piece with as much precision as possible. It has been suggested that the preoccupation with creativity that exists in Western society is maintained by the commercial benefits of musicians distinguishing themselves with a personal identity (Clarke, 2012). Alongside the drive for creativity and individuality has come an upsurge in the number of technologies available for producing and hearing music. These have led to some marked changes in the way music is experienced, and could have either facilitatory or impairing effects on musical creativity.

For example, since audio recording of music performances became possible, more and more of the music that people hear is “disembodied,” comprising only audio, with no visual cues and no possibility of real-time performer-audience interaction. Today, most people have ready access to a vast collection of recordings from a wide range of musical styles. As a result, present-day musicians are exposed to far more musical ideas than would have been the case if they had been born in an era where the only access to music was via live performance. The potentially rich networks of musical knowledge that they have constructed in memory could facilitate their creative musical thinking by providing numerous possibilities for new associations to be made.

On the other hand, over-familiarity with popular interpretations or conventions could constrain either performers' abilities to consider more unusual ideas or listeners' willingness to accept more idiosyncratic performances (see Repp, 1997a). Today, music is everywhere–playing in the background while we work, shop, exercise, travel, and relax–and most people receive a great deal of passive (and often unsought) exposure to certain genres, which might affect their openness to new styles or interpretations. More broadly in the expertise literature, an inverted-U relationship is hypothesized to exist between formal knowledge and creativity, with highly-knowledgeable people sometimes struggling to break away from established frameworks and generate novel ideas (Weisberg, 1999). Future research might investigate collaborative creativity in ensembles comprising professional musicians who are at different stages of their careers.

Some technologies, like music notation software, are designed to make the process of creating music easier and more generally available, including for people who wish to compose collaboratively. These programs usually convert MIDI information into musical scores, so musicians can compose at a keyboard without having to attend to notating their ideas. Alternatively, those who lack technical performance skills can enter notes using a mouse or computer keyboard and hear their ideas played back to them – the ability to play or audiate their own compositions is not necessary. It could be argued that while such programs do simplify the task of composing, they also constrain composers' creativity by minimizing their reliance on imagination and potentially impeding cognitive flexibility. On the other hand, notation software could be seen as a means of composers extending their own working memory capacity. Fewer resources spent maintaining a single idea in working memory means that more resources are available for elaborating on that idea or drawing new associations. Whether the net effect is enhanced or impaired creativity, however, requires some investigation.

Other technologies, like new digital musical interfaces (DMIs), broaden the range of sounds and sound-producing gestures that can be part of a music performance. In some cases, they also reduce the extent to which music performance depends on highly practiced technical skills, making them potential means of music-making for a large number of people. A critical difference between DMIs and traditional instruments concerns how directly gestures and sound output relate. For DMIs, gesture-sound relations are indirect – and sometimes complex: gestures activate electronic signals, which pass through several layers of algorithmic mappings before triggering sound output (Jensenius, 2013). As research has already shown, audience members are sometimes unable to make sense of complex gesture-sound mappings, and show little appreciation for the performed music as a result (Emerson and Egermann, 2017). Do ensemble members also struggle to make sense of each other's gestures when performing with DMIs? When their sound-producing gestures carry little communicative value, what other communication techniques do they use to ensure successful collaboration? Future research should consider whether different mechanisms support collaborative creativity in DMI and traditional instrument contexts.

## 6. Conclusions

Driving our discussion has been the question of how musicians coordinate their performance under conditions that encourage creativity. Despite extensive research into ensemble coordination mechanisms, the literature on music performance has largely avoided the topic of creativity, focusing instead on simplified musical contexts that lack the ambiguity, unpredictability, and variety of real-world music. In recent years, however, the field of music cognition has seen increased interest in studies of music outside the Western classical repertoire e.g., (Freeman and van Troyer, 2011; Marandola, 2014; Clayton, 2017), which has prompted questions about how generalizable our current understanding of coordination processes may be. At the same time, theoretical perspectives have shifted away from treating cognition as individual and internal, moving instead toward embodiment and distributed cognition paradigms. A growing number of studies now focuses on constructs such as group flow, in many cases attempting to develop conceptual models based on investigation of performers' experiences.

Researchers have long shied away from the scientific study of creativity in music performance, presumably because the idea of artistic creativity seems ill-defined and difficult to quantify. I have not ventured into any discussion of how musical creativity or creative abilities should be evaluated, and would argue that the evaluation of creative output is a different issue from describing the underlying processes. The creative processes involved in ensemble performance can be probed objectively and systematically by investigating musician's real-time adaptability and flexibility, testing for differences in behavior or musical output between solo and ensemble playing conditions, monitoring the (multilevelled) audiovisual signals that pass between them, or measuring the patterns of leader-follow influence that come and go throughout a performance.

I have highlighted some potential mechanisms for collaborative creativity, including musical imagery, which could facilitate performance flexibility and adaptability, and multilevelled reflective and prereflective communication processes that could help performers align their constantly-evolving intentions in real-time. I have also discussed the potential importance of empathy in facilitating perspective-taking and coordinating of emotional states. In future research, particular attention should be paid to demystifying concepts such as emergence and flow, perhaps through systematic study of how often they arise and how substantially they affect audience members' perceptions of a performance.

## Author contributions

The author confirms being the sole contributor of this work and approved it for publication.

### Conflict of interest statement

The author declares that the research was conducted in the absence of any commercial or financial relationships that could be construed as a potential conflict of interest.
